# Cathartics in Dysentery

**Published:** 1857-01

**Authors:** O. C. Gibbs

**Affiliations:** Frewsbury, N. Y.


### ART. VI.— *Cathartics in Dysentery.* By O. C. GIBBS, M. D., Frewsbury, N. Y.

At the meeting of the Buffalo Medical Association, Sept. 2d, 1856, as per report of proceedings in the October number of the Buffalo Medical Journal, a discussion took place in regard to the propriety of using cathartics in dysentery; also the *kind* of cathartics best calculated to fulfill the indications in that disease. As this question is fairly before the readers of the Journal above mentioned, we suppose it is open for the expression of opinion or experience, by any of its many readers. Hence, we give expression to a few thoughts, based partially upon our individual experience, and partially upon the generally received opinions in regard to the nature of the affection under consideration. A knowledge of the nature or pathology of any disease, is, perhaps, the surest guide to the appropriate indications of treatment. The public generally, are apt to look upon all diseases accompanied with frequent evacuations from the bowels, as similar at least, if not identical in character. Physicians themselves are not always free from this vagueness of nomenclature. In muco-enteritis, as well as milder forms of mucous irritation, each case is accompanied with a diarrhœa or frequent alvine evacuations, and the public generally do not discriminate between such cases and dysentery, and we have seen physicians not unfrequently, if not guilty of the same error in diagnosis, at least of the same vagueness of nomenclature. Dysentery consists in an inflammation of the mucous membrane of the colon and rectum, and, though the evacuations may be over in ten minutes, yet, except it may be in the incipiency of the disease, they are not fœcal, but consist almost wholly of mucous and blood. Hence, though the griping pains in the abdomen and the tenesmus may be never so great, though the characteristic muco-sanguineous evacuations may be never so frequent, or the straining at stool never so persistent, the case may be accompanied with obstinate constipation. The public generally look upon the frequent bloody evacuations as constituting the whole of the disease, and, consequently, urge the importance of powerful astringents, which, if unadvised by the attending physician, they sometimes clandestinely and injuriously bring to bear upon the disease. But the physician who resorts to them, to the exclusion of evacuants, will certainly have no reason to boast of his success.

Permit us to say, that we do not propose to discuss the nature, cause, or symptoms of dysentery, nor to enter into full details of treatment. We propose only to make a brief expression of our opinion, upon the question under discussion, viz.: the propriety of cathartics in dysentery.

Some authorities have condemned the use of evacuants in dysentery, on the ground of their supposed irritating influence upon the inflamed mucous membrane. But we feel confident that, when the evacuant is judiciously selected, and repeated with due discrimination, and with proper adjuncts, its irritating influence is more fancied than real. The object of the cathartic seems, at first, to be to free the bowels from irritating secretions, and the object of their repetition is, conjoined with the above, to prevent constipation, which is the inevitable sequence of the inflammation and consequent fever. A second, and not less important object to be secured by the evacuant, is to unload the portal veins, thus diminishing congestion in that important circulatory system, and to stimulate the capillary circulation in the liver, which is often sluggish, resulting in a deficient biliary secretion.

In regard to the choice of a cathartic there has been and is a great discrepancy of opinion. Some have advised calomel at first, to be succeeded by castor oil; others have advised castor oil from the first. Rhubarb, compound powder of jalap, cream tartar, epsom salts, rochelle salts, &c., have all had their advocates.

We were formerly in the habit of giving, at first, calomel intimately commingled with rhubarb and a little pulverized opium, and afterwards, whenever an evacuant seemed demanded, gave castor oil with a few drops of laudanum. But recently we have made choice of a different evacuant, and, so far, have been much pleased with the change. In the June number of the *Western Lancet*, for 1855, Dr. D. B. Dorsey communicated the result of twenty years' experience with a cathartic mixture, first proposed to him by Dr. Lemoyne, of Washington, Pa. Summing up his result he said "in a practice, not very limited, in the cities of Wheeling, Va., and Steubenville, O., in the latter of which dysentery prevailed as an epidemic twice or thrice during my residence there, I had the high gratification of seeing all recover who were treated with this remedy from the commencement of the attack." With this high encomium before us, we made trial of the combination in the next case that came under our observation, and with such happy results that, except in young children, we have used it in all dysenteric cases since, with success in all cases.

We quote Dr. Dorsey's formula and directions from the paper above referred to. "Take of saturated solution sulph. magnesia, *seven fluid ounces;* aromatic sulphuric acid, *one fluid ounce*—mix.

"The saturated solution is prepared by dissolving epsom salts in an equal quantity of water, *by weight,* at 60 deg. Fahrenheit. It will be ready for use in eight or ten hours. During that time it should be shaken occasionally.

"The medium dose of this medicine for an adult, is one tablespoonful, delivered with two or three ounces of water, every four to six hours, until it gently moves the bowels. It should be given regularly, and perseveringly, until the bowels are manifestly under its influence, which will be evinced by peculant discharges, abatement of tenesmus, and general feeling of relief. The size of the dose and times of repeating it, must be varied by the practitioner's judgment, according to many circumstances of age, violence and stage of disease, &c. Sometimes it will require two tablespoonfuls of the medicine, every three or four hours; at others a teaspoonful every six or eight hours will be sufficient.

"Accompanying each dose, when the pain and tenesmus are great, one sixth of a grain of sulph. morph. may be given. But this remedy, also, must be varied, both in quantity and frequency of repetition, according to circumstances."

We have seldom or never exceeded tablespoonful doses, and oftener fallen below that. But instead of giving once in four or six hours throughout the twenty-four, we have usually commenced with it in the morning, to be repeated every three hours until it operates, always combined with a small quantity of morphine. This course we repeat every day so long as the indications demand. During the remainder of the twenty-four hours, we give ipecacuanha with morphine, or such other remedies as the circumstances of the case seem to require. It may not be amiss to say here that mercurials are incompatible with the mixture.

The acid doubtless stimulates the capillary circulation in the liver, promoting bilious secretion, while the sulphate of magnesia relieves the portal congestion and frees the bowels from irritating secretions. From the relief which speedily follows its action, to the tormina and tenesmus, greater than that following any other evacuant, we cannot help thinking the acid has a direct sanitary influence upon the inflamed mucous membrane.

With young children, where smallness of dose and pleasantness of taste are always considerations of much importance, the above mixture is decidedly objectionable. The taste is rather disagreeable, and the necessity for diluting the mixture, renders the bulk such as no child will readily take. In such cases we have been in the habit of scorching rhubarb, adding boiling water and extract of hyosciamus, the dose of such proportioned to the age and condition of the patient, sweetening the mixture and flavoring with nitre.

This is to be given in repeated doses in the morning, sufficient to produce a laxative effect, and during the balance of the day we give hydrargyrum cum creta, in small doses, with Dover's powders, or such other medicines as the circumstances of the case may indicate.

